# Comprehensive exosomal microRNA profile and construction of competing endogenous RNA network in autism spectrum disorder: A pilot study

**DOI:** 10.17305/bb.2023.9552

**Published:** 2024-04-01

**Authors:** Sha Zhao, Yan Zhong, Fang Shen, Xinning Cheng, Xiaojuan Qing, Jiamin Liu

**Affiliations:** 1 Department of Child Healthcare, Hunan Children’s Hospital, Changsha, China; 2 University of South China, Hengyang, China; 3 Pediatrics Research Institute of Hunan Province, Hunan Children’s Hospital, Changsha, China

**Keywords:** Autism spectrum disorder (ASD), exosome, microRNA (miRNA), competing endogenous RNA (ceRNA), immune cell infiltration

## Abstract

Exosomes have been demonstrated to exert momentous roles in autism spectrum disorder (ASD). However, few studies have reported a correlation between exosomal microRNAs (miRNAs) and ASD. To date, our understanding of crucial competing endogenous RNA (ceRNA) networks in ASD remains limited. Herein, the exosomal miRNA profile in the peripheral blood of children with ASD and healthy controls was investigated and the level of immune cell infiltration in ASD was evaluated to determine the distribution of immune cell subtypes. Exosomes were isolated from the peripheral blood of ten children with ASD and ten healthy controls, and further identified using transmission electron microscopy and western blot analysis. RNA sequencing was conducted to investigate exosomal miRNA profiles in patients with ASD. The mRNA and circular RNA (circRNA) expression profiles were acquired from the Gene Expression Omnibus (GEO) database. Differentially expressed mRNAs (DEmRNAs), miRNAs (DEmiRNAs), and circRNAs (DEcircRNAs) were identified and ceRNA regulatory networks were constructed. Furthermore, the immune cell infiltration levels in patients with ASD were evaluated. Exosomes were spherical, approximately 100 nm in size, and were confirmed via western blot analysis using exosome-associated markers CD9, CD63, and CD81. Thirty-five DEmRNAs, 63 DEmiRNAs, and 494 DEcircRNAs were identified in patients with ASD. CeRNA regulatory networks, including 6 DEmRNAs, 14 DEmiRNAs, and 86 DEcircRNAs, were established. Correlation analysis indicated that leucine-rich glioma inactivated protein 1 (LGI1) expression was significantly positively correlated with the content of CD8**+** T cells. Our findings may be conducive to offering novel insights into this disease and providing further evidence of transcriptomic abnormalities in ASD.

## Introduction

Autism spectrum disorder (ASD) is a multifactorial disease characterized by impaired social communication and interaction with restricted and repetitive behavior, interests, or activities [1]. Various processes are associated with physiological abnormalities in ASD, including immune dysregulation, oxidative stress, inflammatory responses, and mitochondrial dysfunction [2]. A study conducted by the Centers for Disease Control and Prevention at 11 sites in the United States indicated that the incidence of ASD has increased over the past two decades [3]. Currently, there are no practical therapeutic approaches or specific drugs available for ASD [4]. However, early interventions have been demonstrated to improve language and speech, reduce cognitive decline, and address challenging behavior, consequently, improving outcomes for children with ASD [5]. Hence, there is an urgent need to identify reliable biomarkers for the early detection of ASD.

Exosomes are extracellular vesicles derived from endosomes in the range of ∼40 to 160 nm in diameter (mean ∼100 nm) [6]. They are secreted by diverse cell types and can be separated from various body fluids, including but not limited to, saliva, serum, plasma, and urine [7]. Exosomes contain cargo molecules, including DNA, mRNA, microRNA (miRNA), proteins, and lipids [8]. They participate in multiple biological processes, such as intercellular communication, immune responses, inflammation, cellular homeostasis, and autophagy [9]. Exosomes intrinsically cross biological barriers, including the blood–brain barrier [10], making blood-circulating exosomes valuable for exploring the pathophysiology of brain diseases [7]. Exosome involvement in various pathophysiological conditions, including ASD, has been investigated [11]. However, only a few studies have reported a correlation between exosomal miRNAs and ASD.

The Gene Expression Omnibus (GEO) is a public repository developed and maintained by the National Center for Biotechnology Information that archives and freely distributes comprehensive sets of microarray, next-generation sequencing, and other high-throughput functional genomic data submitted by the scientific community. The GEO database provides the public with circRNA, miRNA, and mRNA data on ASD and is a valuable resource for researchers studying ASD pathogenesis. The competing endogenous RNA (ceRNA) hypothesis proposed in 2011 introduced a new fundamental “language” that describes transcriptional control via miRNA binding sites and miRNA response elements [12]. According to the ceRNA theory, circRNAs act as “miRNA sponges” to regulate target gene transcripts by competing with shared miRNAs. To date, our understanding of pivotal ceRNA networks in ASD remains limited.

In this study, the exosomal miRNA profile in the peripheral blood of children with ASD and healthy controls was investigated. Furthermore, the level of immune cell infiltration in ASD was evaluated to determine the distribution of immune cell subtypes. In this study, we aimed to contribute to the understanding of ASD pathogenesis and provide more evidence for transcriptomic abnormalities in ASD.

## Materials and methods

### Datasets collection

To acquire the mRNA and circRNA expression profiles of ASD, one mRNA dataset (GSE18123) and one circRNA dataset (GSE200197) were downloaded from the GEO database. The GSE18123 dataset contained 31 patients with ASD and 33 controls, whereas GSE200197 had 4 patients with ASD and 4 controls, all analyzing peripheral blood samples. The flowchart of this study is shown in Figure S1.

### Patients and samples

Ten children with ASD (2–4 years old) and 10 normal controls (2–4 years old) were recruited to obtain exosomal miRNA expression profiles. Inclusion criteria for patients with ASD were as follows: (1) meeting the diagnostic criteria for autism according to the Diagnostic and Statistical Manual of Mental Disorders (DSM) criteria (5th edition) [1]; (2) Autism Behavior Scale (ABC) score ≥67; and (3) Childhood Autism Rating Scale (CARS) score ≥30. Patients with organic mental disorders, brain tumors, metabolic diseases, or mental retardation due to other diseases were excluded. Peripheral blood samples (5 mL) were collected from every individual. The demographic information of the participants included in this study is shown in Table S1.

### Exosome isolation

Exosomes were extracted from peripheral blood via supercentrifugation. Blood samples were centrifuged at 1500 *g* for 15 min at 4 ^∘^C to collect supernatants. Supernatants were centrifuged at 10,000 *g* for 30 min at 4 ^∘^C and filtered using a 0.22 µm filter membrane. The supernatant was discarded after centrifugation at 120,000 *g* for 1.5 h at 4 ^∘^C. The precipitate was resuspended in 15 mL phosphate-buffered saline (PBS) at 4 ^∘^C for 100,000 *g* for 1 h, thus leaving exosomes as the pellet formation.

### Transmission electron microscopy

A total of 10 µL suspended droplets of exosomes were adsorbed on ultra-thin carbon-supported copper membrane for 1 min. After removing excess liquid with filter paper, the exosomes were washed with filtered PBS for 5–10 s, followed by staining with 2% uranyl acetate for 5–10 s, and repeated three times. After removing excess uranyl acetate using filter paper, the solution was left to dry. A transmission electron microscope (200 kV) was used for observation and imaging.

### Western blotting

Protein expression levels of exosome-associated markers (CD9, CD63, and CD81) were determined by performing western blot analysis. The total protein concentration of exosomes was quantified using the bicinchoninic acid assay. Protein samples (30 µg) were subjected to 12% sodium dodecyl sulfate-polyacrylamide gel electrophoresis at 80 V for 0.5 h, 120 V for 1 h, and 200 mA for 1.5 h before transfer onto a polyvinylidene fluoride membrane. After blocking with Tris-buffered saline containing 5% dried skim milk for 1 h at room temperature, the membranes were washed with 1×TBST buffer and incubated with primary antibodies overnight at 4 ^∘^C. Finally, the fluorescent signal was quantified using a western blot detection system.

### RNA sequencing

TRIzol LS Reagent (Invitrogen Life Technologies: 10296028) was used to isolate RNA from exosomes. The integrity of the RNA was confirmed using a 2% agarose gel, and the concentration (OD 260) and protein contamination (OD 260/OD 280 ratio) of the total RNA samples were determined using a NanoDrop ND-1000. The quality and concentration of the sequencing library were assessed using an Agilent 2100 bioanalyzer. Sequencing was performed using Illumina NextSeq 500 according to the manufacturer’s protocol.

### Identification of DEmRNAs, DEmiRNAs, and DEcircRNAs

The “edgeR” package in R was used to identify DEmiRNAs and the “limma” package in R was utilized to detect DEmRNAs and DEcircRNAs between ASD and controls with a |log_2_ fold change| > 1 and *P* < 0.05. Gene Set Enrichment Analysis (GSEA) was performed using GSEA (version 4.1.0) to determine enriched gene sets. Hallmark gene sets (c2.cp.kegg.v2022.1.Hs.symbols.gmt) selected in the present study were downloaded from the Molecular Signatures Database. The enrichment results with a *P* value of less than 0.05 were considered statistically significant.

### Construction of ceRNA (DEcircRNA–DEmiRNA–DEmRNA) networks

To better understand the effects of circRNAs on mRNAs mediated by the combination of miRNAs, ceRNA networks were built based on the aforementioned data. Based on the results of differential expression analysis, the targeted DEmRNAs of the DEmiRNAs were predicted using miRWalk. DEcircRNA–DEmiRNA interaction pairs were predicted using circBank. Thereafter, the ceRNA (DEcircRNA–DEmiRNA–DEmRNA) networks were constructed by combining circRNA–miRNA pairs with miRNA–mRNA pairs. Ultimately, Cytoscape was used to visualize the regulatory networks.

### Evaluation of immune cell subtype distribution in ASD

CIBERSORT is useful for high-throughput characterization of diverse cell types from complex tissues that can transform normalized gene expression matrix into a composition of infiltrating immune cells [13]. To evaluate immune cell subtype distribution in ASD, we uploaded gene expression matrix data to CIBERSORT and obtained an immune cell infiltration matrix. The bar plot was used to visualize the proportion of each type of immune cell in different samples. The “corrplot” package was used to draw a correlation heatmap to visualize the correlation of 22 types of infiltrating immune cells. The “ggplot2” package was used to draw violin diagrams to visualize the differences in immune cell infiltration.

### Real-time qPCR (RT-qPCR) validation

Ten children with ASD and ten healthy controls were included in the RT-qPCR analysis. Exosomes were extracted from peripheral blood as described previously. The TRIzol LS Reagent was used to isolate RNA from exosomes. RT-qPCR was performed in a QuantStudio5 Real-time PCR System (Applied Biosystems) with 2× PCR master mix (Arraystar: AS-MR-006-5). Relative gene expression was analyzed using the 2^−ΔΔCT^ method. Human U6 was used as endogenous control. The sequence of primers used in RT-qPCR is presented in Table 1.

**Table 1:** Primers used in RT-qPCR analysis

**Symbol**	**Primers (5’ to 3’)**
*HOXD4*	5’GGGTCACGGACAAGAGCCA3’ 5’GTGTAGTTGGGGTTCACCGAAT3’
*CYP4F11*	5’GGTATTGATGATTTCCTCAAGAAC3’ 5’CAGGTCGTCCCATTCAATCT3’
*F13B*	5’TCTCTCACACCAAAATGTACCAA3’ 5’CGATAAGTTGTTGAATGTGTTTGA3’
*U6*	5’GCTTCGGCAGCACATATACTAAAAT3’ 5’CGCTTCACGAATTTGCGTGTCAT3’
hsa-miR-514b-5p	5’GGGTTCTCAAGAGGGAGGC3’ 5’GTGCGTGTCGTGGAGTCG3’
hsa-miR-521	5’GGCAACGCACTTCCCTTTA3’ 5’GTGCGTGTCGTGGAGTCG3’
hsa-miR-222-5p	5’ATTCCCCTCAGTAGCCAGTGTA3’ 5’GTGCGTGTCGTGGAGTCG3’
hsa-miR-501-5p	5’GGAGAATCCTTTGTCCCTGG3’ 5’GTGCGTGTCGTGGAGTCG3’
hsa-miR-663b	5’AAGGTGGCCCGGCCGT3’ 5’GTGCGTGTCGTGGAGTCG3’

### Validation of mRNAs and miRNAs in the GEO database

The GSE25507 and GSE102741 datasets were downloaded from the GEO database to validate mRNA expression levels. The GSE89596 and GSE67979 datasets were downloaded to validate miRNA expression levels. GSE25507 consisted of 82 children with ASD and 64 controls, and GSE102741 included 13 children with ASD and 39 matched controls. The GSE25507 dataset examined peripheral blood lymphocyte samples and GSE68719 examined postmortem brain tissues. GSE89596 consisted of 30 patients with ASD and 30 controls, and GSE67979 included five patients with ASD and five controls, in which peripheral blood samples were examined.

### Ethical statement

This study was approved by the Medical Ethics Committee of Hunan Children’s Hospital (HCHLL-2020-107). Written informed consent was obtained from the guardians of all the children.

## Results

### Characterization of exosomes

**Figure 1.**
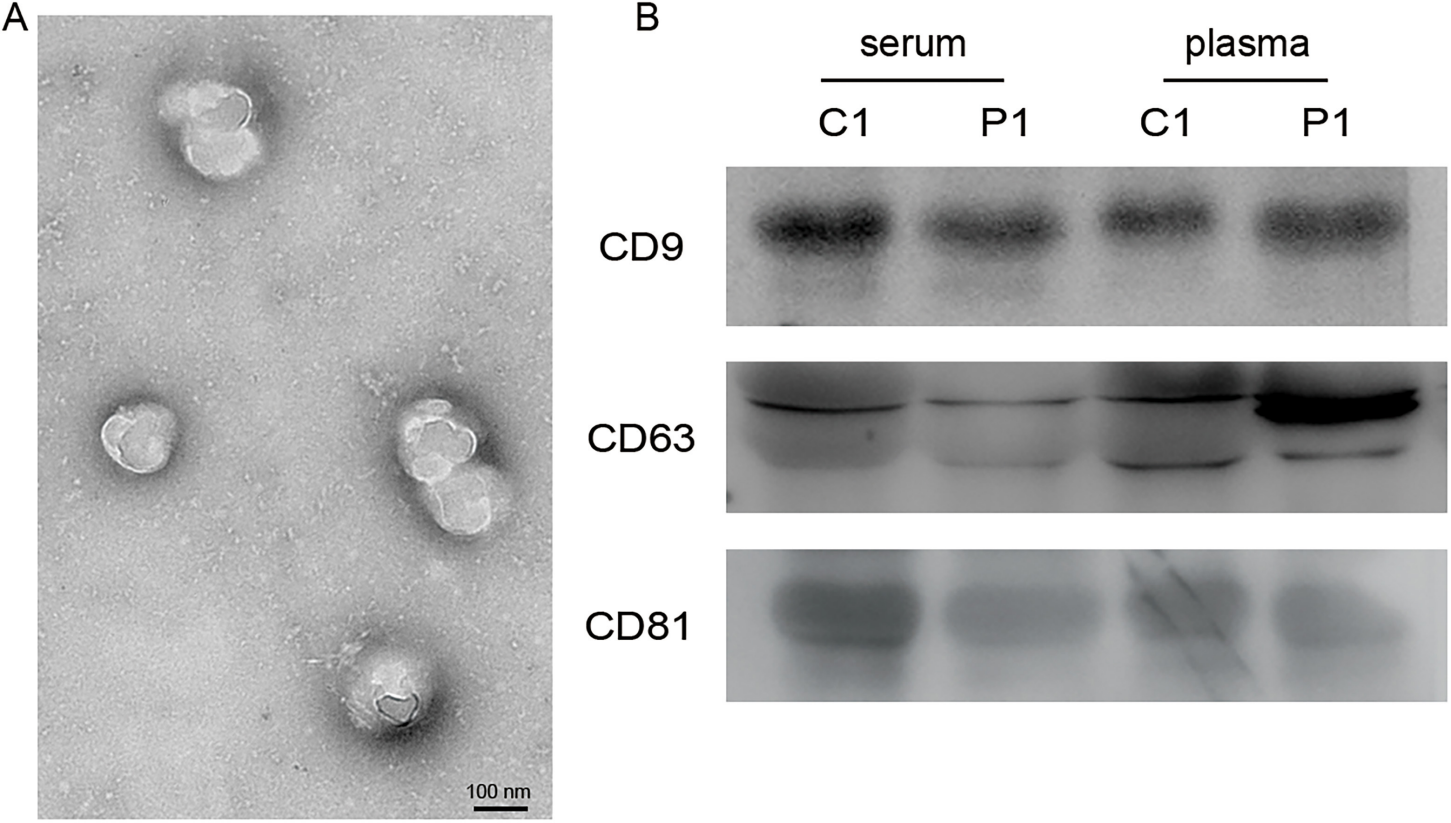
**Characterization of exosomes.** (A) Transmission electron micrographs of exosomes. Scale bar: 100 nm; (B) Western blot analysis of CD9, CD63, and CD81 expression in exosomes. C: Controls; P: Patients.

The morphology of exosomes isolated from peripheral blood was evaluated using transmission electron microscopy. The exosomes were spherical, wrapped in a double-membrane structure, and approximately 100 nm in size (Figure 1A). Exosomes identity was confirmed by quantifying exosome surface markers CD9, CD63, and CD81 via western blot analysis (Figure 1B). The results indicated that both groups of particles expressed these markers at consistent levels, affirming the collected precipitates are exosomes.

**Figure 2.**
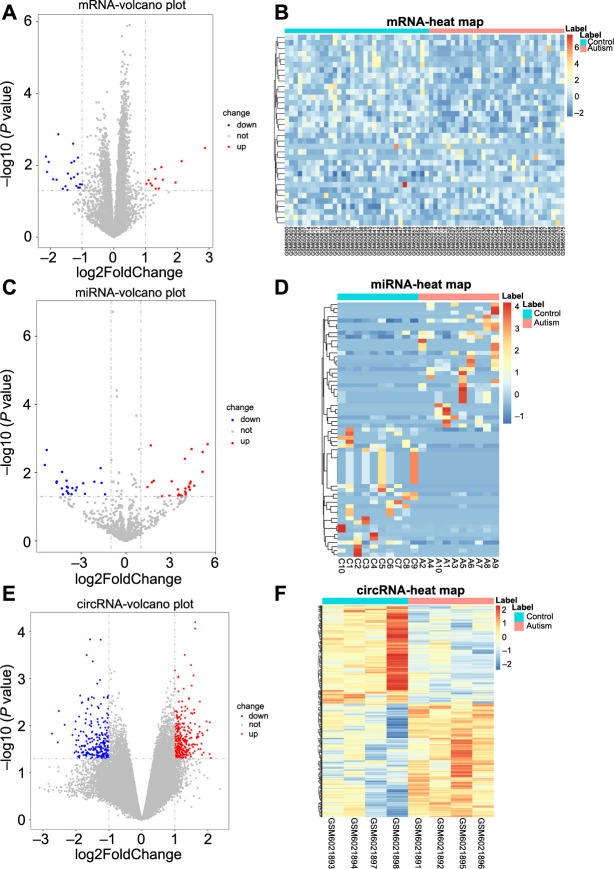
**Identification of DEmRNAs, DEmiRNAs, and DEcircRNAs.** (A) The volcano plot of DEmRNAs; (B) The heatmap of DEmRNAs; (C) The volcano plot of DEmiRNAs; (D) The heatmap of DEmiRNAs; (E) The volcano plot of DEcircRNAs; (F) The heatmap of DEcircRNAs. “down” indicates “downregulation”, “not” indicates “not significant”, “up” indicates “upregulation”. DEmRNA: Differentially expressed mRNA; DEmiRNA: Differentially expressed miRNA; DEcircRNA: Differentially expressed circRNA.

**Figure 3.**
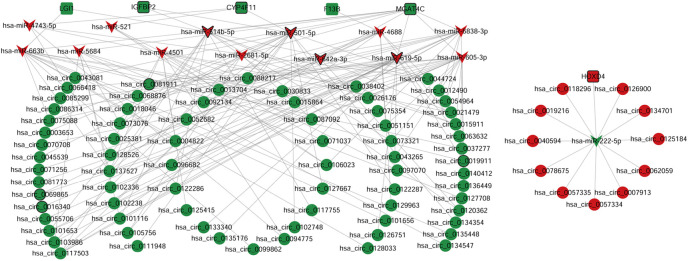
**ceRNA regulatory networks.** The elliptical, inverted triangles, and rectangle nodes indicate DEcircRNAs, DEmiRNAs, and DEmRNAs, respectively. Red and green colors represent upregulation and downregulation, respectively. Nodes with black border are DEmRNAs/DEmiRNAs/DEcircRNAs derived from top 10 up- and downregulated DEmRNAs/DEmiRNAs/DEcircRNAs. DEmRNA: Differentially expressed mRNA; DEmiRNA: Differentially expressed miRNA; DEcircRNA: Differentially expressed circRNA; ceRNA: Competing endogenous RNA.

### Identification of DEmRNAs, DEmiRNAs, and DEcircRNAs

A total of 35 DEmRNAs, 63 DEmiRNAs, and 494 DEcircRNAs were identified in ASD, of which 13 (37.1%), 31 (49.2%), and 265 (53.6%) were upregulated, respectively, whereas the others were downregulated (Figure 2). The GSEA results revealed several gene sets that were significantly upregulated in ASD, such as the Notch signaling pathway, long-term potentiation, and pathways in cancer (Figure S2). CeRNA regulatory networks were constructed to reveal the interactions in circRNA, miRNA, and mRNA in ASD. The miRNAs predicted for the ceRNA networks had conserved binding sites in both circRNAs and mRNAs, as determined by the miRWalk and circBank databases. As shown in Figure 3, ceRNA regulatory networks, including 6 DEmRNAs, 14 DEmiRNAs, and 86 DEcircRNAs, were established. Each component in these pairings exhibited opposite expression patterns. According to the ceRNA hypothesis, miRNAs have contrasting co-expression relationships with mRNA and circRNAs, whereas circRNAs have a positive co-expression relationship with mRNA. These networks may provide a novel and comprehensive perspective on the interactions between miRNA, circRNA, and mRNA in ASD development. Among these circRNAs, hsa_circ_0137527 (degree ═ 6), hsa_circ_0117503 (degree ═ 5), hsa_circ_0013704 (degree ═ 4), hsa_circ_0044724 (degree ═ 4), and hsa_circ_0015911 (degree ═ 4) were the top five DEcircRNAs that interacted with the most DEmiRNAs.

**Figure 4.**
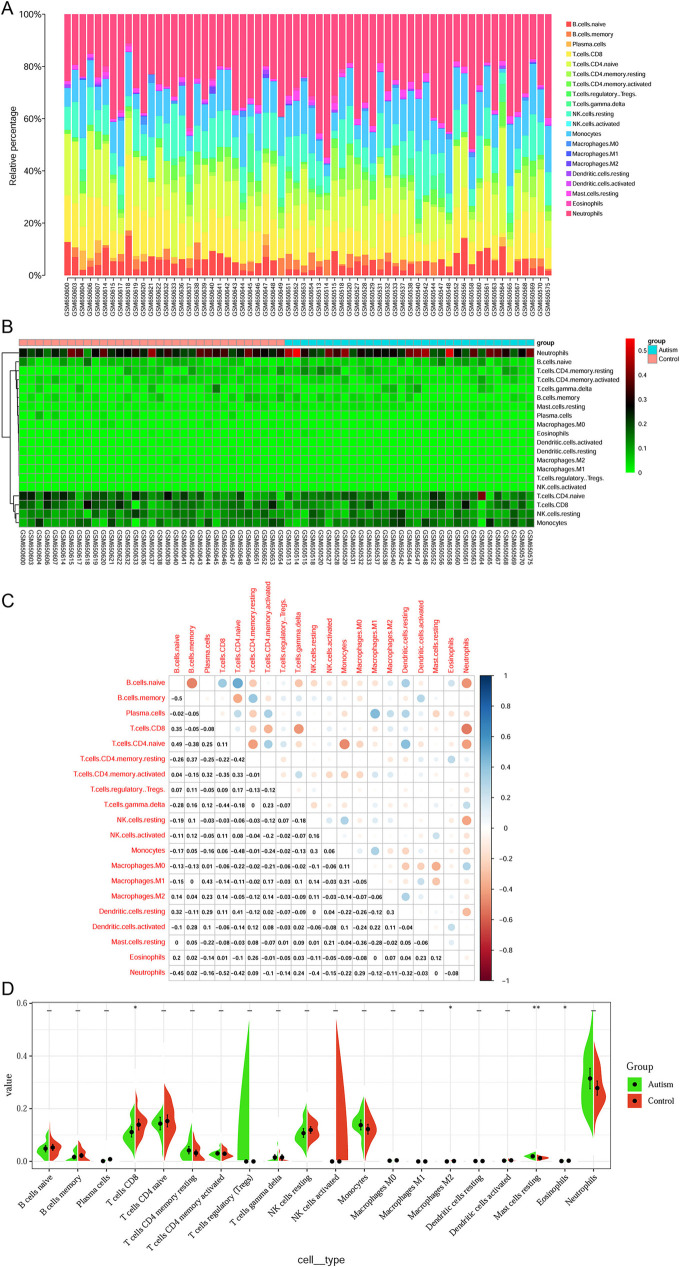
**Immune cell infiltration in ASD.** The composition of immune cells in each sample is shown in a histogram (A) and a heatmap (B). (C) The correlation of immune cells in ASD was evaluated. (D) Violin plot of significantly different immune cell types between ASD and controls. ASD: Autism spectrum disorder.

### Immune cell infiltration in ASD

**Figure 5.**
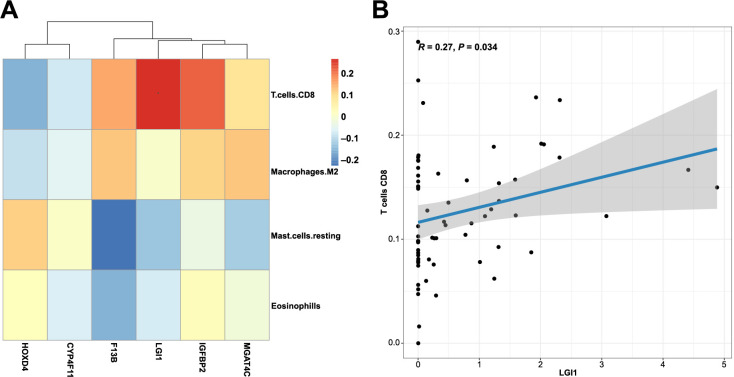
Correlation analysis between hub genes and immune cells.

The CIBERSORT results exhibited 20 subpopulations of immune cells (no percentage of activated mast cells or follicular helper T cells) in patients with ASD (Figure 4A). In the heatmap, immune cells in each sample are shown with normalized absolute abundance (Figure 4B). The results indicated that neutrophils, monocytes, naïve CD4+ T cells, CD8+ T cells, and resting NK cells comprised the majority of these components. Correlation heatmap of the 20 types of immune cells revealed that naïve B cells positively correlated with naïve CD4+ T cells, and negatively correlated with memory B cells. Furthermore, monocytes negatively correlated with naïve CD4+ T cells, and, lastly, CD8+ T cells negatively correlated with neutrophils (Figure 4C). The contents of CD8+ T cells, M2 macrophages, and eosinophils were significantly lower in the ASD group, whereas the content of resting mast cells was significantly higher in the same group (Figure 4D). In addition, to investigate the potential correlation between mRNA expression and immune cell infiltration in ASD, correlation analysis was conducted between the expression of six mRNAs in the ceRNA networks and four types of immune cells (Figure 5A). The results showed that the expression level of Leucine-Rich Glioma Inactivated protein 1 (LGI1) significantly positively correlated with the infiltration level of CD8+ T cells (*P* ═ 0.034, Figure 5B).

### RT-qPCR validation

**Figure 6.**
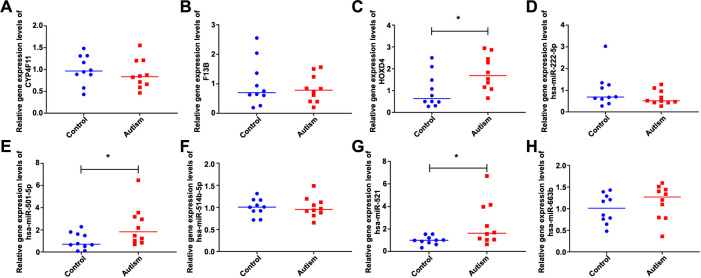
**The results of RT-qPCR validation.** (A) *CYP4F11*; (B) *F13B*; (C) *HOXD4*; (D) hsa-miR-222-5p; (E) hsa-miR-501-5p; (F) hsa-miR-514b-5p; (G) hsa-miR-521; (H) hsa-miR-663b. *P < 0.05. RT-qPCR: Real-time qPCR; HOXD4: Homebox D4.

Three DEmRNAs and five DEmiRNAs were subjected to RT-qPCR validation. As shown in Figure 6, *HOXD4*, hsa-miR-521, hsa-miR-501-5p, and hsa-miR-663b showed upregulated trend, whereas *CYP4F11*, *F13B*, and hsa-miR-222-5p were downregulated in ASD, consistent with the results of our analysis. In particular, *HOXD4*, hsa-miR-521, and hsa-miR-501-5p were significantly upregulated in patients with ASD.

### Validation in the GEO database

Expression levels of 6 DEmRNAs and 14 DEmiRNAs from ceRNA networks were validated using the GEO database. The results indicated that the expression levels of *HOXD4* and *CYP4F11* in GSE25507 and those of *HOXD4* and *F13B* in GSE102741 exhibited trends consistent with our integrated analysis (Figure S3). The expression levels of hsa-miR-521, hsa-miR-501-5p, hsa-miR-222-5p, hsa-miR-2681-5p, and hsa-miR-6838-3p in GSE89596, and those of hsa-miR-222-5p, hsa-miR-663b, hsa-miR-514b-5p, hsa-miR-4688, hsa-miR-4743-5p in GSE67979, exhibited the same trend as RNA sequencing results (Figure S4).

## Discussion

ASD is a major public concern that imposes substantial medical and educational costs on families. Increasing attention has been drawn to the role of peripheral blood-derived exosomes in various diseases, including ASD [4].

The Notch signaling pathway is a well-established developmental pathway in brain morphogenesis [14]. It exerts momentous roles in regulating neural stem cell proliferation, survival, and differentiation during central nervous system development [15]. In addition, dysfunction of the Notch signaling pathway is related to multiple nervous system diseases, including ASD [16]. Recent studies have suggested a potential correlation between Notch signaling pathway dysfunction and ASD [17, 18]. The Notch signaling pathway has been implicated in the occurrence and development of ASD by regulating autophagy and affecting dendritic spine growth in a rat model of ASD [19]. Consistent with previous studies, the Notch signaling pathway was observed to be significantly upregulated in ASD.

Increasing evidence supports crucial roles of the immune system in ASD pathophysiology [20]. Correlation analysis of mRNAs and immune cells suggested that the expression level of LGI1 was significantly positively correlated with CD8+ T cell contents. LGI1 is highly expressed in the central nervous system and may participate in the regulation of neuronal growth. A review indicated that LGI1 may be a major actor of synaptic regulation [21]. Moreover, synaptic dysfunction is associated with ASD pathogenesis and affects typical autistic behavior in patients [22, 23]. IGI1 may participate in ASD pathophysiology by regulating synaptic function. Immunohistochemistry of postmortem brain tissues revealed an increase in CD8+ T cells in ASD [24]. These findings may suggest the great importance of LGI1 and CD8+ T cells in ASD pathogenesis.

*CYP4F11* encodes a member of the cytochrome P450 superfamily. Compared to adjacent tissues, *CYP4F11* overexpression was detected in tumor tissues of Mexican women with breast cancer [25]. Rs1064796 in *CYP4F11* is associated with adverse drug reactions in advanced non-small cell lung cancer [26]. Miao et al. [27] reported that hsa-miR-501-5p is associated with the development of chronic thromboembolic pulmonary hypertension. Yoneda et al. [28] identified hsa-miR-501-5p as a serum biomarker of chronic periodontitis. In addition, hsa-miR-501-5p, a significantly downregulated exosomal miRNA, has been detected in cerebrospinal fluid from congenital hydrocephalus patients [29]. No studies have reported the relationships of hsa_circ_0044724, hsa_circ_0137527, and hsa_circ_0117503 with diseases. In addition to the circRNAs mentioned above, *CYP4F11* and hsa-miR-501-5p were also reported to be abnormally expressed in patients with ASD for the first time in this study. Our findings suggested that the hsa_circ_0044724/hsa_circ_0137527/hsa_circ_0117503/hsa-miR-501-5p/CYP4F11 interaction pairs may function important roles in ASD. However, their exact roles in the underlying biological mechanisms of ASD require further clarification.

Homeobox D4 (*HOXD4*), a member of the homeobox family, is differentially expressed with high tissue specificity and plays crucial roles in morphogenesis. High *HOXD4* expression is associated with poorer prognosis of ovarian cancers and gastric adenocarcinoma [30, 31]. Reduced *HOXD4* expression is associated with unfavorable disease-free survival of kidney renal clear cell carcinoma patients [32]. *HOXD4* expression was associated with the clinical outcomes of glioma patients, making *HOXD4* a promising potential prognostic biomarker for gliomas [33]. Camps et al. [34] reported that hsa-miR-222-5p may function an important role in hypoxia. Gorur et al. [35] reported that hsa-miR-222-5p may serve as an early detection marker for patients with coronary artery disease. Similarly, both *HOXD4* and hsa-miR-222-5p were reported as abnormally expressed in ASD for the first time and were found to interact with each other in ceRNA networks. In addition, 11 circRNAs, such as hsa_circ_0062059, hsa_circ_0007913, and hsa_circ_0057334, were targeted by hsa-miR-222-5p in the ceRNA network, suggesting that these 11 circRNAs may function as hsa-miR-222-5p sponges for the upstream regulation of *HOXD4* in ASD.

A limitation of this study was the small sample size for RNA-seq and RT-qPCR. The lack of significance in the GEO dataset validation may stem from differences among sample types. The sequenced samples were peripheral blood-derived exosomes, whereas the samples in the GEO datasets were derived from the blood or tissue. To date, there is no consensus regarding the relationship between exosomal miRNAs and whole blood miRNAs. Rothschild suggested a correlation between the expression of miRNAs in tumor tissues and blood, implying tumor releases of circulating miRNAs within exosomes [36]. Similarity between miRNA signatures in circulating exosomal miRNAs and originating tumor cells has also been observed in lung adenocarcinoma [37]. To determine whether exosomal miRNAs reflect the miRNA signature in whole blood of patients with complex regional pain syndrome (CRPS), McDonald et al. [38] analyzed miRNAs in exosomes purified from the sera of six patients with CRPS and six healthy controls. The results revealed that 16 of the 18 dysregulated miRNAs in the whole blood of patients with CRPS were detected in human serum-derived exosomes, with five significantly altered, and only one showing the same trend in whole blood and exosomes.

## Conclusion

In this study, we identified 35 DEmRNAs, 63 DEmiRNAs, and 494 DEcircRNAs between patients with ASD and controls, which may be related to the occurrence and development of ASD. Limited overlap was found in the identification of mRNAs, miRNAs, and circRNAs between our study and previous research, owing to sample variations, patient cohort, and technical differences. Although validation of the expression of majority of mRNAs and miRNAs through RT-qPCR and GEO database was not statistically significant, a trend existed, indicating that larger cohorts are necessary. In addition, further experimental verifications are necessary to elucidate the biological functions of these potential markers in patients with ASD, which involve a combining of in vivo and in vitro techniques in future studies.

## Supplemental data

Supplementary data are available at the following link: https://www.bjbms.org/ojs/index.php/bjbms/article/view/9552/2987

## Data Availability

The mRNA (GSE18123, GSE25507, and GSE102741), miRNAs (GSE89596 and GSE67979) and circRNA (GSE200197) expression profiles of ASD are available in the GEO database (https://www.ncbi.nlm.nih.gov/geo/). The raw data of miRNA expression profiles in exosomes derived from ASD patients have been deposited to the GSE222046 dataset (https://www.ncbi.nlm.nih.gov/geo/query/acc.cgi?acc=GSE222046).
